# The incidence and prevalence, diagnosis, and treatment of multiple sclerosis in China: a narrative review

**DOI:** 10.1007/s10072-022-06126-4

**Published:** 2022-05-18

**Authors:** Dongmei Jia, Yu Zhang, Chunsheng Yang

**Affiliations:** 1 Department of Neurology, Tianjin Medical University General Hospital, 154 Anshan Road, Heping District, Tianjin, China; 2 China Medical Affairs, Biogen Inc, Shanghai, China

**Keywords:** Multiple sclerosis, China, Incidence and prevalence, Delayed diagnosis, DMT availability

## Abstract

In 2018, the first list of rare diseases was published by the National Health Council of China, and multiple sclerosis (MS) was included in this list. Since then, the Chinese government and neurologists have made efforts to improve the clinical outcomes of patients with MS. During last few years, the incidence of MS in China was also investigated. The early and accurate diagnosis of MS was improved due to the application and promotion of magnetic resonance imaging and new diagnosis criteria. The market for and medical insurance access to disease-modifying therapies (DMTs) has been greatly accelerated, which has provided more treatment options and improved clinical outcomes for patients with MS, as well as reduced treatment cost. The pattern of MS in China is gradually changing, from delayed to early diagnosis, and from no treatment to treatment with DMTs during remission. This narrative review aimed to summarize an update to the status of MS in China, including incidence and prevalence, diagnosis, and available treatments. This would help to better understand the diagnosis and treatment gap between mainland China and other Asian regions, demonstrating the necessity of accurate diagnosis and optimized treatment of MS in China.

## Introduction

Multiple sclerosis (MS) is a chronic inflammatory and immune-mediated demyelinating disease of the central nervous system [1]. Reports from Atlas of MS estimated that there were 2.8 million people with MS in the world in 2020, a number that has increased by 0.5 million compared with 2013 [2, 3]. In China, the first list of rare diseases was published by the National Health Council of China until 2018, and MS was included in this list [4]. In the past 2 decades, several classes of disease-modifying therapies (DMTs) were approved by the US Food and Drug Administration (FDA) and European Medicines Agency (EMA) [5], changing the dynamics of MS and making MS a more manageable disease [6]. However, in China, interferon β injection was the only marketed DMT before 2018 [7]. Teriflunomide was the first oral DMT approved by China’s National Medical Products Administration (NMPA) in 2018, 6 years later than the FDA approval [7, 8]. This narrative review aims to summarize the current status of MS in China, including incidence/prevalence, diagnosis, and treatment, to characterize the diagnosis and treatment gap between China and other Asian countries, demonstrating the necessity of accurate diagnosis and optimized treatment of MS in China.

## Incidence and prevalence of MS in China

In China, the first MS case was recorded by Peking Union Medical College Hospital in 1926, and the first autopsy report of MS was made by Shanghai Huashan Hospital in 1957 [9]. The first nationwide study reported that the incidence of MS in China was 0.235 per 100,000 person-years in 2018 based on an administrative database of the National Hospital Quality Monitoring System [10]. The incidence of MS in China was higher in high-latitude areas [10], which was similar to reports from other countries [11–13].

There are no nationwide studies assessing the general prevalence of MS in China, although several reports have been published that describe the prevalence of MS in different regions (Table 1) [14–17]. From these reports, the number of patients with MS in mainland China seems to have not changed significantly in the past 3 decades and is significantly lower compared with Europe and the USA (over 100/100,000 people) [18]. One of the regional reports, a door-to-door survey in 1992, revealed that the prevalence of MS was 2.1/100,000 people in an autonomous county, Yunnan Province [14]. Cheng et al. reported that the crude prevalence of MS was 1.39/100,000 people in Shanghai in 2005 [15]. In 2013, the estimated prevalence rate of MS was 5.2/100,000 people in Shandong Province [17]. Another population-based study using national medical insurance databases of six provinces in mainland China reported that the urban prevalence rate was 2.91, 2.32, and 2.44 per 100,000 people in 2012, 2015, and 2016, respectively [16]. Li et al. reported that the prevalence rate of MS was 2.39/100,000 people after comprehensive analysis of three prevalence studies [19]. In summary, based on these reports, the prevalence rate of MS ranges from one to five per 100,000 people in mainland China.

**Table 1:** Summary of MS prevalence in mainland China from different studies

Year	Prevalence*	*n*^#^	Regions	Data source	Ref
1992	2.1	424,628	An autonomous county in Yunnan Province	Door-to-door survey	[14]
2005	1.39	8,860,000	Shanghai City	Population-based survey	[15]
2013	5.2	95,792,719	Shandong Province	Hospitalized data	[17]
2012	2.91	195,440,000	Six provinces (Zhejiang, Inner Mongolia, Liaoning, Qinghai, Guangdong, and Chongqing)	National medical insurance databases	[16]
2015	2.32				
2016	2.44				
2021	2.39	–	–	Comprehensive analysis of three prevalence studies	[19]

*MS* multiple sclerosis, *Ref.* reference

^*^Prevalence, data shown as per 100,000 population; ^#^*n*, number of people covered

Unlike in mainland China, the number of patients with MS has greatly increased in Hong Kong and Taiwan during the past 40 years. The prevalence rate of MS in Hong Kong was 0.88 and 0.77 per 100,000 people in 1989 and 2002, respectively [20, 21]. In 2006, the prevalence rose to 4.8 per 100,000 people in Hong Kong due to the application of magnetic resonance imaging (MRI) and implementation of a hospital computerized surveillance system [22]. Similarly, the prevalence rate of MS among Chinese individuals was 0.8/100,000 population in northern Taiwan in 1976 [23]. According to a population-based epidemiological study, the prevalence rate of MS was 2.96/100,000 in 2005 in Taiwan [24] and rose to 6.69/100,000 in 2015 [25].

In other Asian countries like Japan, the prevalence of MS has also gradually increased in the past years [26, 27]. The prevalence rate was two to four per 100,000 people in the 1960s and one to four per 100,000 people in the 1980s [28, 29]. In 2003, the crude prevalence was 7.7/100,000 people [30]. Based on an analysis of a health insurance claims database, the prevalence rate of MS rose from 15/100,000 to 19/100,000 people between 2011 and 2015 in Japan [27]. The prevalence of MS in northern Japan was 8.1, 12.6, 16.2, and 18.6 per 100,000 people in 2001, 2006, 2011, and 2016, respectively [26].

In summary, the prevalence rate of MS did not change greatly in mainland China over the last decades, whereas it has gradually increased in Hong Kong, Taiwan, and Japan. Several factors may have contributed to this phenomenon: (1) the risk of developing MS might be lower in mainland China, which should be verified by nationwide prevalence studies; (2) the diagnosis of MS is delayed because of lack of disease awareness among neurologists and radiologists; (3) patients with MS are under-diagnosed because they have a lack of disease knowledge and no access to specialized MS centers; and (4) there is no nationwide registry system for MS in China.

In 2021, the China National Registry of Central Nervous System Inflammatory Demyelinating Diseases (CNRIDD; NCT05154370, www.clinicaltrials.gov) was initiated and focused on the establishment of a national, multicenter disease registry to provide disease-related information on patients with inflammatory demyelinating diseases (IDDs) in China. This project will generate a comprehensive picture about IDD including MS, providing the nationwide incidence and prevalence, diagnosis, treatment and facilitating management of patients with IDD in China in the future.

## Delayed diagnosis of MS in mainland China

The currently used MS diagnosis criteria is the 2017 McDonald criteria, according to Expert Consensus for the Diagnosis and Treatment of Multiple Sclerosis in China 2018 (MS Expert Consensus) [31, 32], which indicates that the diagnosis criteria in China are consistent with international guidelines. The updated diagnosis criteria increased the sensitivity and accuracy of MS diagnosis in China.

According to evaluation results from Suzhou, China, the 2005 McDonald criteria were more sensitive than Poser criteria (90.2% vs. 72.0%) for MS diagnosis [33]. In Chinese patients with clinically isolated syndrome (CIS), the 2017 McDonald criteria had a higher sensitivity (75.0% vs. 14.6%) and higher accuracy (67.7% vs. 36.9%) compared with the 2010 version [34]. Therefore, the 2017 McDonald criteria are suitable for the early diagnosis of MS in Chinese patients. These findings may also be one of the explanations for the previous low prevalence of MS in mainland China, because the diagnosis criteria (Poser and McDonald 2010) with low sensitivity and accuracy could cause under-diagnosis in patients with MS.

Additionally, data suggest that MS diagnosis in Chinese patients is delayed for 5–10 years since the first disease onset, especially in smaller, nonspecialized centers. It was found that the mean age at MS diagnosis was 36 years, whereas the mean age at MS onset was 31 years, based on the analysis from Beijing Tiantan Hospital, a leading MS center in China [35]. Based on the nationwide hospital-based study, the mean age at MS diagnosis was 45.3 years old in tertiary hospitals of China [10]. In two review papers published in 2009, the age of MS onset was around 30 years in Chinese patients with MS [9, 36], which was similar to findings from other studies [37, 38]. This means that there was a delay of more than 10 years for MS diagnosis and/or a high proportion of misdiagnosis or missed diagnosis of MS.

One of the major reasons for the delayed diagnosis is seemingly a lack of knowledge about the disease among patients with MS. In the Multiple Sclerosis Patient Survival Report 2021 in China (MS Survival Report 2021), 1540 patients with MS from 41 centers in mainland China were included and completed the questionnaires prepared for this survival survey, including diagnosis, treatment, prognosis, and quality of life. In this report, 24.5% of the patients with MS did not seek clinicians’ help immediately upon initial onset of MS and there was a 1.33-year delay from MS onset to the first hospital visit. Among patients, 96.3% had never heard of MS at the time of diagnosis in this report [39], which was consistent with the results from the MS Social Survey 2020 and other smaller studies [40, 41].

Another factor for delayed MS diagnosis is misdiagnosis of MS. According to the MS Survival Report 2021, 50.3% of patients were initially misdiagnosed or under-diagnosed at the first hospital visit, with a 2.27-year delay of accurate diagnosis [39]. Based on the MS Social Survey 2020, the average interval was around 1 year from the first hospital visit to final diagnosis of MS; in one patient, the longest interval was 22 years [40]. A multicenter analysis of top hospitals in China showed 55.1% of patients did not get a confirmed diagnosis of MS during the first visit, with a delayed duration of 0.9 years [41], which did not change in the last few years [39].

Taken together, although the currently used MS diagnosis criteria in China are the 2017 McDonald criteria, which are recommended by various guidelines and consensus for early diagnosis, the accurate diagnosis of MS in China still seems considerably delayed. Most Chinese neurologists and radiologists are up-to-date in employing diagnostic tools for MS, including the use of recent diagnostic criteria and MRI. However, patients with MS generally lack insight on the disease, thus highlighting the importance of strengthening patient education and raising awareness of MS in order to promote early and accurate diagnosis of MS. It is likely that the real prevalence of MS might be much higher than current published data due to misdiagnosis and underdiagnosis of MS. Hopefully, this situation will be improved with the implementation of the CNRIDD program.

## DMT availability for MS in mainland China

**Fig. 1:**
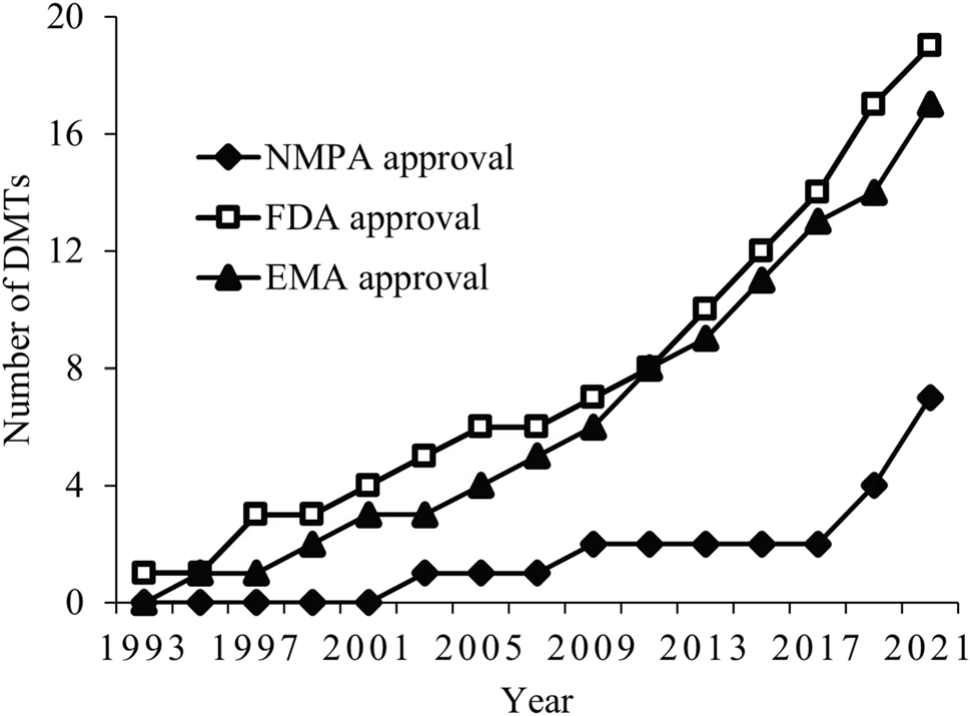
The number of DMTs approved by FDA, EMA, and NMPA. *DMT*, disease-modified therapy; *EMA*, European Medicines Agency; *FDA*, US Food and Drug Administration; *NMPA*, China’s National Medical Products Administration

An increasing number of new MS drugs have been approved and marketed in the last 2 decades (Fig. 1) [5], and DMTs have become standard of care for patients with MS in many countries [42–45]. However, there are only seven DMTs approved by NMPA (Table 2) [46]. Before 2018, there was only one marketed DMT (interferon-beta) [7]; most patients were prescribed corticosteroids, immunosuppressants, immunoglobins, and interferons [36]. These medications were recommended in the MS Expert Consensus (2014) recommendations for the management of MS [47].

**Table 2:** MS drug approval time among NMPA, FDA, and EMA

	NMPA	FDA	EMA
Interferon β-1a	2003	1995	1997
Interferon β-1b	2009	1993	1995
Teriflunomide	2018	2012	2013
Fingolimod	2019	2010	2011
Siponimod	2020	2019	2020
Dimethyl fumarate	2021	2013	2014
Fampridine*	2021	2010	2011
Ofatumumab	2021	2020	2021

*DMT* disease-modifying therapy, *EMA* European Medicines Agency, *FDA* US Food and Drug Administration, *MS* multiple sclerosis, *NMPA* China’s National Medical Products Administration

^*^Fampridine, fampridine is a drug to improve walking impairments in patients with MS, but not a DMT

Since the first list of rare diseases was published in 2018 [4], the Chinese government has made efforts to accelerate medication access to improve the prognosis of patients with rare disease. In 2018, the first oral DMT (teriflunomide) was approved by the NMPA [5]. Siponimod, fingolimod, dimethyl fumarate and ofatumumab were also approved within 3 years [48–50], providing more treatment options for Chinese patients with MS. Furthermore, efforts have also been made to accelerate the access of medical insurance to reduce treatment cost of MS by National Healthcare Security Administration in China. In 2019, interferon β-1a and teriflunomide [51], and in 2020, fingolimod and siponimod, were included into national medical insurance [52]. In addition to DMTs, fampridine, the only FDA- and EMA-approved drug to improve walking impairments in patients with MS [53, 54] was granted accelerated approval in May 2021 [55] and was included in national medical insurance in December 2021 [56]. The fast access to market and medical insurance indicates that increasing attention has been paid to the management of clinical symptoms to improve the quality of life in patients with MS in China.

Although the Chinese government has made attempts to provide more cost-effective DMTs, the proportion of treated patients is still low [39, 40, 46]. According to the MS Survival Report 2021, 58% of patients with MS did not receive any medication during MS remission [39], which was similar to the MS Survival Report 2018 with 60% of untreated patients [57]. It has been noted that DMT coverage in patients with MS has almost doubled within 3 years, from 10 to 18% between 2018 and 2020 [39, 57]; however, this rate was still lower than Australia and Germany, where the DMT penetrance was 64% and 57%, respectively [58, 59].

As for the patients who did not receive any treatment, approximately 30% did not think it was necessary to receive a maintenance treatment during remission to prevent MS relapse [39, 57]. Around 30% of patients did not receive treatment because the cost of medication was high [39, 57], and 59% discontinued treatment because they could not afford the cost of medication [40].

In summary, treatment options are still limited for patients with MS in China, but attempts are being made to provide DMTs and medical insurance inclusion to provide more DMTs with reduced cost for these patients. It was noted that the approval interval of DMTs was greatly shortened from over 10 years to 1 year between FDA and NMPA (Table 2). In addition to providing access to DMTs, both neurologists and patients should be educated to increase the DMT coverage during MS remission, which will improve the prognosis of MS.

## Future perspectives

To improve outcomes for patients with MS in China, and to raise the awareness of MS in China, measures should be taken to improve the early and accurate diagnosis and patient knowledge of MS. Moreover, treatment options are very limited in mainland China, with only four oral DMTs approved by the NMPA out of the nine approved by the FDA. All four of these oral DMTs were approved by NMPA since 2018, when MS was included in the first list of rare diseases. The Chinese government has made efforts to shorten the approval interval between NMPA and FDA; thus, it is expected that an increasing number of DMTs will be introduced to China in the near future, which might help to reduce MS disease activity and improve the quality of life in patients with MS.

The inclusion of medical insurance should be encouraged to reduce the cost of MS treatment, which will benefit more patients with MS. In addition to disease treatments, more attention should be paid to the management of clinical symptoms, such as cognition, fatigue, and mental health, to improve the quality of life in MS patients.
